# Supplementary material for: Humans prioritize walking efficiency or walking stability based on environmental risk
Source: PLoS One. 2023 Apr 7;18(4):e0284278. doi: 10.1371/journal.pone.0284278 (PMC10081767; doi:10.1371/journal.pone.0284278)
Supplement: Attachment — Submitted filename: Response to reviewers.docx [file pone.0284278.s001.docx]

We thank the reviewers for their insightful comments. We have addressed these, and our responses are below.

We have updated the ethics statement as required by PlosOne.

Reviewer #1: 

This manuscript describes a study undertaken to test the test the hypothesis that humans manipulate passive anterior-posterior(AP) stability via active selection of step length to either achieve energy-efficient gait or to improve stability when balance is threatened. The authors utilize the extrapolated center of mass concept, and the UCM concept to show that the margin of stability during unobstructed gait is stabilized, and that during (and before) obstacle crossing, subjects choose a more careful strategy (which the authors argue is more energy efficient). Overall, this is a cleverly designed study, that is written up in a very readable fashion. However, I do have some suggestions and comments.

Major comments
1) Concerning terminology; most of the time, the authors talk about “stability”, but sometimes they also use the term “balance” (for instance, in the title). However, it is unclear how these concepts are different according to the authors. It may be good to define these terms (and maybe only use stability or balance?)

RESPONSE: We agree that the use of different terms is not ideal. We have adopted the reviewer’s advice and adopted ‘stability’ throughout – including in the title of the paper.

2) Regarding “passive anterior posterior stability”; how passive is this? Given that the authors argue that it is controlled, is it passive? I understand what the authors mean (i.e. that if you place your feet there, you can exploit passive dynamics), but is the stability really passive then?

RESPONSE:

Our claim is that humans control the initial conditions of the passive pendular dynamics. By actively positioning the foot humans select the initial position, and by modulating pushoff, humans set the initial velocity. These initial conditions then determine the passive pendular dynamics of the subsequent swing phase. Actively selecting the initial conditions does not eliminate the passive component of the subsequent swing phase. MOSap quantifies this passive component of the resulting movement.

Furthermore, we included in the Limitations section a discussion of the issues with MOSap. There, we talk about the difficulty in inferring overall gait stability from MOSap.

3) The link where the data is provided is not accessible. I would also suggest to put the data in a more permanent repository, such as Zenodo, Figshare, Datadryad. All of these have the advantage that 1) they also provide a DOI (so the dataset is citable), and 2) they are searchable by major search engines (so the data is findable).

RESPONSE: We apologize that the link we shared during the initial submission did not work. But we have now published our data and code. The doi is available with this revision.

4) Regarding the margin of stability, and statements made about this on lines 163/164: This is ONLY true if there would be no collision loss at foot placement (which there of course is), and when there would be no push off (or, if collision loss and push off would be equal). See work on the Foot Placement Estimator (Millard et al., 2009, DOI: 10.1115/1.4000193) which does not have this problem (as it takes the collision into account) and Bruijn & van Dieën 2018(doi: 10.1098/rsif.2017.0816.) for a discussion on this. Hence, the authors need to be more carefull with such statements.

RESPONSE: We agree. Thank you for pointing this out. We have updated the interpretation of MOSap. We now adopt the view of Hak et al (2019; <https://doi.org/10.1016/j.jbiomech.2018.12.037>) and Raffegeau et al (2022 <https://doi.org/10.1016/j.exger.2022.111710>). We have also mentioned in the Limitations section some challenges associated with interpreting MOSap.

5) I think the whole story that this must be actively controlled (in the discussion), is well, only strong for the obstacle crossing. I think Patil et al (2019) (doi: 10.1016/j.jbiomech.2019.109375) would tend to disagree with this. I think at best, the argument can be made that active control CONTRIBUTES.

RESPONSE: Agreed. This is what we are saying: that active control contributes. Please note the way we start that section: “It is unlikely that passive mechanics of the gait cycle ALONE can explain the positive synergy indices that we observed. It is indeed likely that mechanics contribute; for example…”

We have added another sentence at the start of this section to make this explicit, and we conclude this argument by mentioning again that passive and active processes contribute. That should leave no doubt about our position in this matter.

However, we would respectfully disagree that these arguments are applicable for the obstacle crossing task only. The synergy indices we obtained point to active control even for unobstructed gait. One point of clarification is that we regard the involvement of spinal loops as active control, and we are suggesting that spinal involvement in unobstructed gait is reflected in the positive synergy indices. We are aware that the current thinking, driven by the seminal work by Art Kuo, is that locomotor control in the AP direction is passive. But please note that even Kuo in his papers relegates AP control to ‘spinal or low-level control’ (cf. Bauby and Kuo, doi: [10.1016/s0021-9290(00)00101-9](https://doi.org/10.1016/s0021-9290(00)00101-9)). So, AP locomotor control is not entirely passive – devoid of any and all modulation of muscle activity.

Minor comments:
1) Line 43; regarding push off adjustments; see also a recent preprint from our group; (<https://doi.org/10.1101/2022.03.14.484283>)

RESPONSE: Thank you for pointing out this work to us. We have cited the preprint in the revision.

2) Line 179; the authors refer to equation 1, but that is not present?

RESPONSE: The equation is present; perhaps the equation number (which is also present) gets buried in the text.

3) I do really like the first part of the results (well done), and the figure that goes with it. However, I had some trouble figuring out which subplot was what; maybe include a small title above each subplot?

RESPONSE: Thank you for the suggestion. We have made this change.

4) Discussion, lines 330-331:” with a corresponding correction in step length so that the MOSAP itself is invariant at each heel contact (supporting H1).” This is not fully true, as there is some variance. I would suggest to change to : ”with a corresponding correction in step length so that the MOSAP itself is MORE OR LESS invariant at each heel contact (supporting H1).”

RESPONSE: Thank you for the suggestion. We have made this change.

5) Line 391; I think not only Dingwell argues this, but also much other work, for instance of van Leeuwen et al., Reimann et al, Rankin et al, etc etc.

RESPONSE: We agree. We altered this paragraph to include the more correct references.

Reviewer #2:

The authors have used the AP margin of stability to understand whether control is energy vs stability focussed in differing environments, level vs perturbation of an obstacle. Additionally they use the UCM to determine if CoM and step length co-vary across these tasks in order maintain MOSAP. They find changes in MOSAP when crossing the obstacle, suggesting changes to improve stability as compared to energy efficient gait. Also the UCM indicated co-variation to maintain specific values of MOSap that were step dependent.

In general the paper was well written, but I found there was insufficient background and justification provided for particularly the main aim of determine whether energy or stability were maintained across different tasks. On this note previous literature has also drawn attention to the difficulty of understanding what anterior-posterior margin of stability means within a gait context, which has not been addressed within this manuscript where MoS has been the key parameter. Additionally, there are elements of the methodology which remain unclear, and potentially may bias the results. For example why are level steps separated into categories – there should be no differences in the level steps and so should be categorised within a single category. More specific comments can be found below.

RESPONSE: We address two points raised here: (1) interpretation of MOSap, and (2) the statistical model used.

First, it is correct that the interpretation of MOSap is problematic, as mentioned by Bruijn et al (2013) (http://dx.doi.org/10.1098/rsif.2012.0999). Thank you for pointing this out to us. We have modified our interpretation of MOSap based on a more thorough review of the literature.

We now adopt the view of Hak et al (2019; <https://doi.org/10.1016/j.jbiomech.2018.12.037>) and Raffegeau et al (2022; <https://doi.org/10.1016/j.exger.2022.111710>). More anterior location of the relative to the BOS boundary indicates (1) more efficient gait, and (2) lower passive dynamic stability in the anterior direction since a forward perturbation will more likely lead to a forward fall.

We have also discussed in the Limitations section some challenges associated with interpreting MOSap.

Second, we altered our statistical model to account for the reviewer’s point. We found no changes in our overall conclusions (the F, p values, dofs, etc. were different, of course). We chose to retain our current statistical model because we think that it allows for a more nuanced description of the findings. However, we have added a statement in the limitations section that acknowledges the alternate stats approach. We also mention that our data shows effects of gait initiation and termination. Therefore, all steps for the unobstructed trials are not really equivalent. This is another reason for retaining our original statistical approach.

Below are more specific comments on the manuscript.

Introduction
In general, I find this section overly long without cohesion to enable a reader to follow the justifications for the aims. Most specifically when setting up the aims for “energy efficient vs stability” as priorities of control this is unclearly stated and needs to be further justified and explained.

Line 38-41: please ensure you use appropriate references for statements based on prior publications

Line 43-46: As mentioned above, this is unclear. Further explanation needs to be provided to the reader on why they may be less able to resist perturbation etc… Separately on this note, past work on perturbations has suggested that can still be stable due to adaptations available such as step length/width adjustments to accommodate perturbations and prevent failure. This is an example of where your justification for the energy vs stability argument could do with re-writing to make very clear to the reader where this is coming from. Additionally line 46-47, this depends on the controller in use, or are you referring to a purely passive gait here? Please clarify.

Line 48-53: Recommend incorporating into above paragraphs and clearing up the argument to provide greater clarity to the reader on your justification

RESPONSE: We have reworked the introduction entirely to address all of these criticisms.

Line 56-57: not necessarily true, in unanticipated perturbations, close to dead-beat control has been shown.

RESPONSE: We agree. We have tempered the claim and say that changes in behavior ‘usually’ occur over time.

Line 63: first used here, so please explain what you mean by “proactive stability”

RESPONSE: Our phrasing was indeed confusing. We simply mean proactive changes in stability, which, in turn means changes in stability during the approach steps before the gait kinematics change substantially during the crossing steps. The phrase ‘proactive stability’ does not appear in the revised introduction.

Line 68-69: based on past literature I feel that more reasoning for why Margin of stability is the appropriate metric to use in this study? Past work (i.e. Bruijn et al 2013), has particularly highlighted that it is unclear as to what MOSAP shows and its meaning within the greater control context. In addition to this there needs to more set up to explain to the reader why negative indicated energy efficient walking – this is unclear, as only indicated unstable gait. Line 73 in line with this would maintaining MOSAP negative and remaining unstable be considered a strategy?

RESPONSE: We agree that MOSap needed to be better interpreted. We have altered the text to clarify this issue.

We now utilize the thinking that more anterior location of the XcoM indicates simultaneously (1) greater utilization of existing kinetic energy to continue walking (so, efficient walking; Hak et al. 2019), and (2) a higher likelihood of a forward fall in case of a trip (so, lower passive stability; Hak et al. 2019, Raffegeau et al. 2022). We do not call gait unstable based on MOSap values anymore; this indeed seems problematic.

As to the appropriateness – we acknowledge that there are other measures that can be used (e.g., foot placement estimator, as suggested by reviewer 1). However, we think that MOSap provides some relevant, valid, and useful information. Furthermore, MOS is popular (likely because of the ease of measurement); since it is going to be used by researchers, offering better interpretations of the measure will be beneficial. We thank the reviewer in helping us improve our manuscript in this regard.

Furthermore, we have included in the Introduction a justification for using this measure.

Line 79-82: unclear as previous hypothesis is that MOSAP will remain invariant so surely these two statements oppose one another?

RESPONSE: We respectfully disagree. We are quite explicit in mentioning that MOSap for a given step number will be consistent for a specific step, but its value will be different across steps for obstructed gait. Nevertheless, we have edited the text to improve clarity.

Line 95: You have not clearly defined the meaning of positive and negative MOSAP values earlier in the introduction

RESPONSE: In the reworked introduction, this distinction is not made. Furthermore, in the revision, this distinction is not critical. We suggest that the gait should not be classified as stable/unstable based on the sign of MOSap. We only say that stability is directly proportional to MOSap.

Line 102: Explain why stabilisation would be made more difficult

RESPONSE: We do not have any additional rationale for this expectation. We think that signal-dependent noise is sufficient for posing this hypothesis. If the actuators are noisy, controlling the output will be more difficult.

Methods:

Line 113: What sample size did you determine for power? Was this achieved?

RESPONSE: We did not perform power analysis for this study. Our sample size was constrained by the resources at our disposal. However, given the significant effects and (mostly) large effect sizes, we think that the issue of power is not that relevant for this study.

Line 115-116: What do you mean by vision normal or corrected-to-normal? Were some of the participants visually impaired?

RESPONSE: ‘Corrected to normal’ simply means that participants wore their prescription glasses or lenses. No one was visually impaired during our experiment.

Line 122: was the obstacle tipped then, if so how many times, and were these trials included?

RESPONSE: Thank you for pointing this out. Across all trials and all participants, the obstacle was contacted and tipped 12 times out of 3360 trials (0.4 % of trials). We did not include the contact trials in the analysis. We have mentioned this detail in Section 2.3.

Line 124: why were all participants required to lead obstacle crossing with their right leg? Some participants would have potentially found this “unnatural” as would be more comfortable leading with their left leg. This also forces them to “target” with a specific leg at a specific time and has potential biomechanical implications on the results as well. How was this worded to the participants?

RESPONSE: Thank you for pointing this out. We instructed all participants to start walking with the left leg. We adjusted the distance between the starting location and the obstacle such that the participants naturally crossed the target with the right leg first.

We also assessed footedness using the Waterloo footedness questionnaire and found that all participants were right-leg dominant. This will further mitigate any concern with ‘unnatural’ stepping. We have added this information in the Methods and results sections.

Line 125: were these randomised or will there potentially be a “learnt” behaviour within the results as well?

RESPONSE: We did not randomize the trials. Participants first performed the unobstructed task and then the obstructed task. We have added this information in the Methods section.

We do not think that learning is an issue in this familiar task that we perform quite frequently. Furthermore, a few obstacle crossing trials were required to identify the starting position for each participant so that they took five steps to reach the obstacle and naturally crossed the obstacle with the right foot first. We assumed that these initial trials (not included in the analysis) were sufficient to achieve any learning, if it is required at all.

Line 131: It is very unclear to me as to why level terrain data have been split into different categories. This generates false categorisation, particularly as each level step (unless the level terrain condition had differences), is same. These should be categorised within one grouping and not split into different group and then the analyses should reflect this as well.

RESPONSE: This is a good point. We assume that by ‘level terrain data’, the reviewer is referencing the unobstructed task in the question above.

To address this issue, we combined the data for the unobstructed task across all the steps (since they are identical in principle), and then performed one-way ANOVA with 7 levels (one unobstructed step and six steps for the obstructed condition) for all outcome variables. We performed all pair-wise comparisons with Tukey-Kramer adjustments. All these new results were compatible with our results with the two-way ANOVAs. Our interpretation of the results and overall conclusions remain the same.

We prefer to retain the two-way analyses because it allows for a more nuanced description on a step-by-step basis. We think that the proactive adjustments are important and wish to emphasize these with the 2-way analysis. Furthermore, as we had indicated in the limitations section, we see some effects of gait initiation and termination in our data. Therefore, all steps are not exactly equivalent for the unobstructed trials. Pooling data across steps for the unobstructed trials may therefore not be ideal.

We have included all this information in the Limitations section.

Line 142: What were the selection criteria for the 15 good trials? Did all participants have 15 good trials? How do you account for speed within your analyses, as speed will affect the kinematics? Were variables normalised to account for anthropometric effects?

RESPONSE: Please note that we say ‘trials with good kinematic data’, not ‘good trials’. This simply means that all markers had fewer than 15 consecutive frames of missing data for the trial.

Yes, all participants had 15 good trials. For participants that had more than 15 good trials, we selected 15 trials at random.

Please note that speed is used to compute XcoM and thus the MOSap. It is also one of the outcome variables that we analyze. We do not think there is any other relevant and interesting way to consider speed.

We did not normalize our outcomes for any other anthropometric parameter. In figure 4 (now figure 3), the MOSap data is provided in meter to be consistent with the common practice in the MOS literature. Since the other outcomes in figure 4 (now figure 3) are the components of MOSap, we decided to report them in meters as well.

In Figure 3 (now figure 4), we could compute the variance components (Vucm and Vort) in terms of statures^2 instead of m^2 by dividing various distances by participant height. We have not performed this computation because we do not expect that the results will be any different given the large effect sizes. More importantly, the key outcome variable – the synergy index – is unitless because it is a ratio of the variance components. So it is not affected at all by computing the variance components in statures^2. To be consistent throughout the paper, we chose to report variances in m^2.

Line 145: Was the AP position of the heel used to denote stance initiation or also end stance? If the latter as well how did you account for the fact that in rear-foot running the heel will begin to move forwards before the end of the stance phase?

RESPONSE: Please note that all our analyses are performed at only one time instant – heel contact. We did not identify end of stance. Also, we are not investigating running. So, we are somewhat confused by the second question.

Line 156: why ankle and not foot markers? At what time point was leg length calculated at?

RESPONSE: We perform all computations at heel contact. This is mentioned in the caption of Figure 1 and it is mentioned multiple times in Section 2.3. Using the ankle joint center for recording leg length is established method in clinical practice (Magee & Manske, 2020). We have assumed the ankle joint center is the pivot of the inverted pendulum model – this is common in the literature on MOS (Peebles et al <https://doi.org/10.1016/j.jbiomech.2016.11.009>; Bosse et al https://doi.org/10.1016/j.humov.2012.05.003).

The use of some other marker on the foot could also be justified. But we do not expect a different choice of pivot to alter any of our results and interpretations, again, given the large effect sizes that we observed.

Magee, D. J., & Manske, R. C. (2020). Orthopedic physical assessment-E-Book. Elsevier Health Sciences.

Line 157: Unclear as to what the average leg length was used for? Why was an average used and not the leg length in the particular trial when XCoM was being calculated?

RESPONSE: That is correct. The average leg length was used to compute XcoM. We have added this clarification in the revision. The variation in leg length across trials was minimal. We did compute the XcoM and MOS using the leg lengths for individual trials and obtained minor changes in MOS values. The statistical results remained unaltered. We chose to average the leg length to be consistent with published literature (e.g., McAndrew Young et al. <https://doi.org/10.1016/j.jbiomech.2011.12.027>; Fallahtafti et al. doi:10.3390/biomechanics1010009; Peebles et al. http://dx.doi.org/10.1016/j.jbiomech.2017.08.023).

Line 162-168: Many assumptions given here without theoretical background support. Suggest use the literature to help provide your reasoning, but also include further details to explain your justifications.

RESPONSE: We have altered this text to reflect the correct interpretation of MOSap. We also provide references supporting our interpretation of MOSap. In particular, this interpretation has been adopted by others (Hak et al., 2019; Raggegeau et al. 2022).

Line 202: Why not between tasks as well?

RESPONSE: We of course test the difference in the synergy index between tasks – that is one of our main hypotheses. This is done using the two-way ANOVAs described in the text that follows immediately. The t tests are used to first identify whether a synergy exists, as stated in the beginning of the sentence.

Line 208-210: Unclear as to why in one analysis participant is the random effect whilst in the other you use trials as the random effect. Please provide further details.

RESPONSE: Thank you for pointing this out. There were errors in our description that we have fixed.

The UCM analysis quantifies the structure in the variance. Therefore, data from 15 trials is used to obtain three numbers: the synergy index, Vucm and Vort. These three outcomes are analyzed with separate ANOVAs where participant is the random effect.

For the other variables, we use the data from all 15 trials (rather than the across-trial averages) in our statistical model. Therefore, the trial number is nested within each participant, and it is a random effect.

We have corrected two typos in this section. Earlier, we had mentioned that all ANOVAs have nested random effects, that the same stats model was used to analyze the UCM outcomes and step length.

First, the UCM outcomes do not have a nested random effect. As explained above, the participant is the random effect for these ANOVAs. Second, as explained above, step length was analyzed using all 15 data points.

Results
Please make sure that within the results section you are only guiding the reader through your results, rather than describing the figures such as in lines 217-220. These details are for figure legends only. This will make your results section more cohesive and condensed. Most of 217-230 is not necessary as are more clearly presented in the lower paragraphs. As mentioned above in the methods section, my biggest concern is the separation of the level steps into categories as well – this is mis-leading and means comparisons between obstacle and level data are not true comparisons but only comparing across partial sets of level data.

RESPONSE: We would like to respectfully disagree here. We think that an overview of the results is beneficial. We also note that Reviewer 1 found this text useful. Therefore, we have decided to retain this section. We have added clarification that detailed results follow in the subsequent subsections.

We do not think that the stats results are misleading. As explained earlier, performing the stats in a different way does not change our results/conclusions. Also, in both the two-way and the alternate one-way ANOVAs, similar number of pairwise comparisons would be performed. So, this would not influence the results either. In the current stats model we perform either 22 (for variables with six levels across six steps) or 28 (for variables with seven levels across seven foot placements) pairwise comparisons. For the alternate one-way ANOVAs, we performed either 21 (when step length was the independent variable) or 28 (when foot placement was the independent variable) pairwise comparisons. From this point of view, the results of our two-way ANOVA are just as robust as those obtained from the alternate one-way ANOVAs.

Finally, we have mentioned the alternate statistical approach in the Limitations section.

Line 225: Please provide the evidence for this.

RESPONSE: Please note that this is an overview of the results. The evidence is provided in Sections 3.1 onwards. We added a sentence at the beginning of this paragraph that the detailed statistics are provided next.

Line 251: suggest re-word to clearly show only Step 0 and +1 were different

RESPONSE: We have clarified this point.

Line 276: Recommend moving the Margin of stability section above the UCM as this follows earlier writings in the introduction etc… Suggest keeping the same order throughout the manuscript

RESPONSE: Thank you for this suggestion. We have rearranged the material in the revised manuscript. The sequence of the hypotheses was switched, the result sections 3.1 and 3.2 were switched, and Discussion sections 4.1 and 4.2 were also switched. Figures 3 and 4 were also switched. Now, the MOSap material appears first, and the UCM/synergy material appears afterwards.

Line 284-285: Does not appear the case in 4A based on the letters.

RESPONSE: Thank you for pointing this out this typo. The text now reads: MOSAP was higher (**more** stable) for fp-1 relative to the two preceding foot placements.

Line 293: This would be clearer if shown as a % between rear and front

RESPONSE: We agree that this data can also be presented as % of step length. However, we selected the current units (m) for Figure 4B (now 3B) because this is the number that enters the calculation of MOS. We think that the text is sufficiently clear.

Line 313: Not reflected in the letters used to depict significant differences

RESPONSE: We altered the text to clarify that the step length is higher for Step-1 compared to Step-3, as seen in Figure 4D (now 3D).

Line 314-315: recommend rewording as it is unclear

RESPONSE: We have edited the text to improve clarity.

Discussion
Rather than stating elements “support H?”, it would be useful to remind the reader what each hypothesis was as currently it is difficult to follow which section answer which element

RESPONSE: We have mentioned our hypotheses in this paragraph.

Line 334-335: Did not change for all steps? Also if level steps were considered as one category rather than randomly split, the differences would only likely occur in FP+1 and FP+2 and potentially FP-1.

RESPONSE: Correct, MOSap did not change for all steps. The sentence does not claim that it did. Therefore, we have retained the sentence. Please note that this is an overview of the results. Adding details makes it difficult to appreciate the overall picture.

Also, the alternate statistical approach (one-way ANOVA) did not change our overall story. Certainly, the specific pairwise-differences were different: MOSap for unobstructed step was smaller than that for fp-4, fp+1 and fp+3. However, MOSap for obstructed steps dropped from fp-4 to fp-3 and fp-2, and then increased for fp-1 and fp+1. The comparisons across obstructed steps show the proactive change in MOSap – which is the main finding of the study obtained from our statistical approach.

Also, as mentioned earlier, all steps of the unobstructed trials are not equivalent; the initial step/fps show signs of gait initiation. This information is included in the Limitations section.

Line 338: please see the introduction comments on justifying your efficiency vs stability argument

RESPONSE: Our revised introduction addresses this point.

Line 357: There are also a multitude of different potential strategies that can be used in order to cross a step.

RESPONSE: If the reviewer can mention other strategies, we will certainly evaluate how those would affect our basic point that higher muscle activations come with more noise. It is difficult to imagine any strategy that does not require an increase in the activation of some muscles to lift the foot over the obstacle.

Line 358-360: It is unclear from figures 2 & 3 whether these shifts are similar in size and direction to provide this direct compensation results in MOSAP stabilisation.

RESPONSE: This is a good observation. Such visual patterns are tricky to spot. Keep in mind that the Vucm and Vort data are across-participant averages, and the y-axes scales are different for the figures 3B and 3C (now 4B and 4C). The Vort values are an order of magnitude smaller than Vucm values, so we had to shrink the y range for Vort to make the across-task and -step patterns visible.

It is best to trust the algebra. The compensation is reflected in positive values of the synergy index for the crossing step. This is evident from the formula for DeltaV, where the numerator is the difference between Vucm and Vort. Increase in Vort is obvious in Figure 4C. But the increase is not sufficient to overwhelm Vucm and yield a negative synergy index (DeltaV).

Line 364-374: Please re-word this section as currently confusing. Purely passive dynamics have been shown to be sufficient to control gait on level terrain, therefore spinal feedback isn’t always necessary.

RESPONSE: We have revised this paragraph to improve clarity.

We do not think that human gait is possible without spinal feedback. This may be true in some physical robots or in-silico models. But even for robots (e.g., those of T McGeer), that the basin of attraction was quite small.

It is unlikely that humans rely entirely on passive dynamics even in the most benign environments given the noise in their neuromuscular system. In fact, there is strong evidence that stretch reflexes are active during human locomotion. Dietz et al. (1987, J. Physiol. (1987), 386, pp. 149-163) showed that during walking, perturbation responses in the leg muscles arise over short latencies (65-85 ms), indicating spinal reflexes at work. Furthermore, it has specifically been argued that spinal reflex help in stabilization of limb trajectories during human walking (Zehr and Stein, 1998, Progress in Neurobiology Vol. 58, pp. 185 to 205). It is also generally accepted that there is a two-way interaction between the central pattern generators and peripheral sensory feedback from the limbs during human locomotion (Loeb 1989, https://www.jstor.org/stable/1311186; Zehr and Stein, 1998; Takakusaki 2013, DOI: 10.1002/mds.25669; Dietz 2003, doi:10.1016/S1388-2457(03)00120-2). Feldman (2021, https://doi.org/10.1016/j.clinph.2021.08.016) outlines a theory of locomotion control that is based upon the recruitment of muscles via stretch reflexes. The details of this theory are being fleshed out, (cf. Ramadan et al 2022, <https://doi.org/10.1038/s41598-022-11102-1>).

And finally, persons with spinal cord injuries cannot walk. It is clear that spinal reflexes are central to our current understanding of human locomotor control.

We have added a few of these details to support our view on the involvement of spinal reflexes.

Line 376: changes are in FP+1 and +2, by your definition these are crossing steps not approach steps.

RESPONSE: Yes, fp+1 and fp+2 are part of crossing steps. However, fp-1 is part of an approach step. So, the first comparison in the parentheses justifies our inclusion of ‘approach step’ in the preceding text. The second comparison across fp+1 and fp+2 is an example of changes across crossing steps.

Line 380: Please further clarify how you linking the positive synergy index to supraspinal mechanism involvement.

RESPONSE: Please note that involvement of supra-spinal mechanisms in synergy modulation is generally accepted based on a body of evidence from the study of neurological patients. We have added this information to strengthen our point. We also clarify that supra-spinal mechanisms could contribute to the synergy by modulating the gains on the spinal reflexes.

Line 393-396: If this is the case why did you not investigate the ML direction alongside the AP?

RESPONSE: We did not investigate MOSml because it is not the focus of the present work. The manuscript is complex and detailed as it is.

Line 403: but this still was not positive, and difficult to interpret what greater or smaller values actually mean for the stability of gait.

RESPONSE: In our revised interpretation of the MOSap, the sign of MOSap is not critical. Higher MOSap means greater stability and vice-versa, as we explain in the introduction. The rationale is that overall stability of human gait must include the contributions of active processes. So, classifying gait as unstable overall based on passive mechanics alone is untenable. Nevertheless, the passive mechanics will influence the overall stability: when MOSap is low, forward perturbations will be harder to reject, indicating lower stability. Note that this interpretation has been suggested before (e.g., Hak et al 2019; Raffageau et al., 2022). Therefore, we can interpret statistically significant differences in MOSap even when there is no change in its sign.

Line 405: increased step length only seen in 2 steps, not across all.

RESPONSE: Yes, this is correct. The text does not suggest otherwise. Please note the wording ‘for some steps’ at the end of the sentence.

Line 410: But return to “normal gait” appears to be relatively fast as by FP+3 in figure 4 all values were within the level bounds.

RESPONSE: We agree. Recovery back to normal gait is faster than the change to the unique crossing steps. We refrain from commenting on this aspect since this is not the focus of the paper.

Line 424-427: As mentioned several times above, I find this argument has not been sufficiently justified earlier in the manscript. Currently what the results do show is a return to “normal” level mechanics within a step of the perturbation – so close to a dead-beat controller.

RESPONSE: We have now altered the introduction to justify the tradeoff between stability and efficiency.

Again, we are not focused on the recovery; investigating that phase of the obstacle-crossing task may be interesting on its own. We mention this in the Limitations section. Also, we are not sure how a dead-beat controller is relevant to the point we are making – that stability is enhanced at the cost of efficiency. If the MOSap has changed a certain way at heel contact, it has definite consequences for the energy consumption. If the return to ‘normal’ walking happens quickly with a dead-beat controller, that would still take control effort.

Line 462-463: Unclear how the methods could be valuable in understanding age and pathology – needs to be expanded if to be included.

RESPONSE: In the extension to the limitations section, we have briefly mentioned how our findings could serve as a baseline to study older or patient populations. Therefore, we have retained this sentence.

Figures
Figure 2: Step IDs need to be included on each subplot and will make it clearer to the reader. Large X & Y axes muddle the plots – suggest label each axes without the use of the large axes which are suggesting they are set within a larger plot. Ellipse description in the legend is unclear as to what they are depicting, and also is the flatness and direct correlation to the synergy index? If so then the approximate values represented should be shown in the figure.

RESPONSE: We have included step IDs in each subplot as recommended. We have also eliminated the large axes, and we have included X and Y labels sparingly to control the clutter in the figure.

The text in the caption mentions clearly: the flatness of the ellipse represents the synergy index with flatter ellipse indicating higher synergy index etc. We are not sure how the text can be clearer. This figure is meant to provide an overview. We decided not to include the synergy index values. We think this detail is not essential and would increase clutter.

Figures 3, 4: Letters used to show differences between steps, very unclear. If saying A is different from B but not AB then this is confusing as the letter A is still within the letters provided! Recommend using the same letters to show difference such that e.g. FP-2 = A; FP-1 = AB and FP+2 = B, shows that -2 and -1 are different to one another and -1 and +2 are different to one another

RESPONSE: Please consider that the suggested method is not really any simpler or clearer than the method we have used. A reader not familiar with the suggested method will have to work out and interpret the pair-wise differences anyway. This means that the difficulty that the reviewer faced using our method is not really reduced by the alternative. We think that the difficulty is in interpreting so many differences, not in the method used to depict the differences.

In the method that we use, A is not different from AB because ‘A’ appears in ‘AB’. This is clearly mentioned in the figure captions. We chose to retain this method; at least some readers will be familiar with this approach given that it is used in scientific publications and in the output provided by statistical software (e.g., SAS).
